# Dissecting regional variability in Pyrazinamide prescribing practices for tuberculosis treatment in Japan

**DOI:** 10.1016/j.jctube.2024.100497

**Published:** 2024-11-22

**Authors:** Nobuaki Kobayashi, Hiromi Matsumoto, Takeshi Kaneko

**Affiliations:** Department of Pulmonology, Yokohama City University Graduate School of Medicine, 3-9 Fukuura, Kanazawa-ku, Yokohama, Japan

**Keywords:** Regional variation, Pyrazinamide, Tuberculosis, Prescribing patterns, Elderly patients, Respiratory specialists

## Abstract

**Objectives:**

To investigate regional variations in pyrazinamide (PZA) prescribing across Japan’s 47 prefectures and associated influential factors.

**Methods:**

This study utilized the Standardized Claim Ratio (SCR) for PZA from Japan’s National Database of Health Insurance Claims in 2018. Pearson’s correlation coefficients assessed relationships between SCR and tuberculosis (TB) incidence, patient characteristics (age, liver disease), and healthcare resources (specialists, TB beds). Multiple regression analysis identified independent predictors of SCR.

**Results:**

Median SCR for PZA was 90.0 (range 40.2–187.1), with a 3-fold difference between top and bottom prefectures. In univariate analysis, SCR correlated positively with TB incidence (r = 0.42), respiratory/infectious disease/TB specialists, and negatively with elderly TB patients (r = -0.33) and liver disease per TB case. Multiple regression revealed higher SCR associated with higher TB incidence (β = 0.44, p < 0.001), lower elderly patients (β = -0.33, p = 0.005), and more respiratory specialists (β = 0.41, p < 0.001).

**Conclusions:**

Regional PZA prescription patterns are multifaceted, significantly influenced by TB prevalence, elderly patient ratios, and the availability of respiratory specialists. To enhance PZA prescribing conformity and TB management, fostering respiratory expertise across Japan is imperative.

## 1 Introduction

Tuberculosis (TB), caused by the *Mycobacterium TB complex*, is a disease that stands as a leading cause of death from infectious diseases among adults worldwide [1]. According to the World Health Organization's report in 2022, TB had been seeing over 6 million new infections annually up until 2019, with a significant decrease in 2020 due to the impact of COVID-19, despite a previously increasing trend each year. It is estimated that between 1 and 3 million patients die from TB each year [2]. A particular threat to public health is drug-resistant strains of TB. Past reports have suggested that without rapid diagnosis and treatment of rifampicin (RFP)-resistant TB, the incidence of TB will continue to increase [3], [4], [5]. Therefore, to prevent the emergence of drug-resistant TB, the foundation of TB treatment is multidrug therapy. The most basic treatment involves administering a combination of isoniazid (INH), rifampicin, pyrazinamide (PZA), and ethambutol (EB) for two months, followed by INH and RFP for four months, once drug susceptibility is confirmed [6], [7]. Randomized controlled trials have demonstrated the desirability of daily administration (as opposed to intermittent) of all drugs during this period [8].

In Japan, the historical context surrounding PZA use in elderly patients warrants particular attention. While the current treatment guidelines advocate a standard quadruple regimen including PZA for tuberculosis treatment, previous editions of the Japanese Society for Tuberculosis guidelines (prior to 2018) explicitly recommended avoiding PZA in patients aged 80 years and older due to concerns about severe hepatotoxicity [9]. This age-specific recommendation, which was unique to Japan's clinical practice, significantly influenced nationwide prescribing patterns, with studies demonstrating PZA exclusion rates exceeding 75 % in patients aged 80–84 years during 2009–2014 [10]. Although this guideline was revised in 2018 based on emerging evidence supporting PZA's safety in elderly patients, there are concerns that the historical recommendation's influence continues to shape current clinical practice, potentially leading to suboptimal treatment regimens in this vulnerable population.

Therefore, this study aims to elucidate regional variations in PZA utilization and identify associated factors influencing prescribing practices. Understanding the key drivers behind differential PZA use can guide efforts to promote more consistent adoption of guideline-recommended quadruple regimens, optimizing TB treatment outcomes nationwide.

## 2 Method

### 2.1 National health insurance system

In Japan, all citizens are covered by public health insurance, and patients receive between 10 % and 30 % of the total amount of medical care they receive, depending on their annual income [11]. The remaining amount is paid when each medical institution files a claim with the Medical Fee Examination Organization in each of the 47 prefectures and is deemed appropriate [12]. Reimbursement for medical care, testing, and treatment is uniform throughout the country, and medical institutions must provide medical care at this fixed price. Furthermore, the Infectious Disease Control Law stipulates that 95 % of the medical cost for TB treatment shall be borne by the prefectural government and only 5 % by the patient [13].

The National Database of Health Insurance Claims and Specific Health (NDB) was established in 2009 based on the “Act on Securing Medical Care for the Elderly. The NDB Open Data has eight major categories: ”Medical Practice,“ ”Dental Practice,“ ”Dental Injuries and Diseases,“ and ”Dental Injuries and Diseases,“ and is used to collect and analyze data on medical practices, dental practices, and dental injuries and illnesses. The NDB Open Data publishes data in eight major categories: ”medical practice,“ ”dental practice,“ ”dental injuries and diseases,“ ”drugs,“ ”specific insured medical materials,“ ”specific medical checkups (test values),“ ”specific medical checkups (standard questionnaire),“ and ”dispensing practices (from FY 2020),“ and is freely available to anyone [14], [15], [16].

### 2.2 Study design

This study employed a prefecture-level ecological study. Standardized Claim Ratio (SCR) data generated from the NDB, which includes almost all insurance practice data, was used to examine differences in prescribing trends of PZA among prefectures. We also examined the impact of the profile of TB patients (prevalence of TB, proportion of elderly TB patients, and prevalence of liver disease) and medical resources (specialists and number of TB beds) on prescribing trends in each prefecture. We selected 80 years as the age cutoff based on historical guidelines from the Japanese Society for Tuberculosis, which advised against PZA use in this age group. This age-specific guidance was grounded in concerns over hepatotoxicity risks, leading to widespread clinical adoption and ongoing influence on prescribing practices.

### 2.3 Standardized claim ratio

In this study, the Standardized Claim Ratio (SCR) was employed as an outcome to compare the amount of PZA prescribed in each prefecture after adjusting for the effects of age and gender.

The SCR is calculated by the following formula [17]

$$\mathrm{SCR}=\;\frac{\mathrm{Observed}\;number\;of\;claims}{\mathrm{Expected}\;number\;of\;claims}\;\times \;100$$

$$=\frac{\Sigma \;Observed\;number\;of\;claims\;of\;each\;age\;group\;i\;\times \;100}{\Sigma \;Number\;of\;each\;age\;group\;i\;of\;standard\;population\;\times \;Claim\;rate\;of\;age\;group\;i}$$

The SCR is normalized to have a national average of 100, which serves as a reference point for comparing prescribing patterns across prefectures. Values above 100 indicate higher-than-average prescribing rates, while values below 100 indicate lower-than-average rates, after adjusting for age and gender distributions. This standardization approach enables direct comparisons between prefectures by eliminating the confounding effects of demographic variations. For example, an SCR of 150 indicates that the prefecture's PZA prescribing rate is 50 % higher than the national average, while an SCR of 50 indicates it is 50 % lower.

### 2.4 Data Source

All publicly available data were used in this study: SCR was obtained from the Cabinet Office, the number of respiratory specialists was obtained from the Japanese Respiratory Society, the number of TB patients and the number of TB patients over 80 years old were obtained from the Epidemiological Information Center of the TB Research Institute of the Japan Society for TB Prevention, the number of TB beds was obtained from the Ministry of Health, Labor and Welfare, and the number of liver disease patients was obtained from a patient survey.

### 2.5 Statistical analysis

JMP Pro 16 software (SAS Institute) was used for statistical analysis.

Descriptive statistics were used to compare SCRs for each prefecture. To examine the correlation between SCR and each item, single regression analysis was performed to obtain Pearson's correlation coefficient. Furthermore, multiple regression analysis was performed with the clinically important items as explanatory variables and SCR as the objective variable.

Pearson's correlation coefficient r was defined as no correlation when |r|<0.2, weak correlation when 0.2<|r|<0.4, moderate correlation when 0.4<|r|<0.7, and strong correlation when 0.7<|r| [18].

### 2.6 Ethical considerations

The NDB Open Data on which this study was based is anonymized medical claims data compiled and published by the Japanese Ministry of Health, Labour and Welfare (MHLW), and the information is de-identified. Therefore, the need for informed consent was waived.

3 Result

### 3.1 Tuberculosis patient distribution across prefectures in Japan

**Fig. 1:**
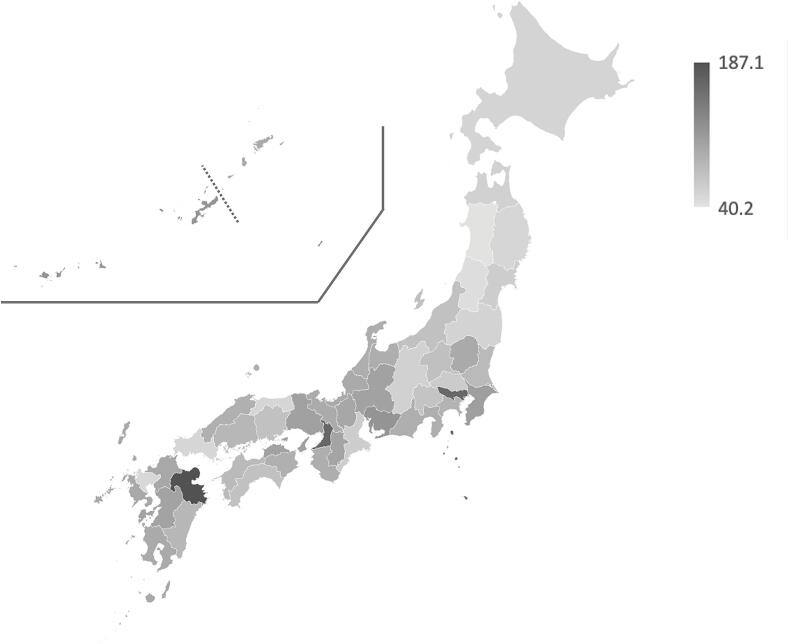

In analyzing the distribution of TB patients across Japanese prefectures in 2018, Table 1 and the corresponding Fig. 1 reveal significant regional disparities. The prefecture with the highest number of TB patients was Tokyo, with 1,382, and the prefecture with the lowest prefectures with low numbers was Tottori, with 51. The median number of patients was 156. The highest prevalence of TB was in Nagasaki Prefecture (16.6/100,000 population) and the lowest in Kanagawa Prefecture (3.6/100,000 population) with a median of 9.8/100,000 population.

**Table 1:** Numbers of patients,amount of prescribed drugs, and SCRs in each prefecture.

Prefecture	Population (×ten thousands people)	Number of TB patients	Prescription amount of PZA (g)	SCR
Hokkaido	529	296	14,235	53.5
Aomori	126	127	4,358	57.4
Iwate	124	105	3,619	52.3
Miyagi	232	91	6,476	60.3
Akita	98	74	NA	40.2
Yamagata	109	65	NA	44.7
Fukushima	186	183	5,694	54.2
Ibaraki	288	304	11,283	79.4
Tochigi	195	172	9,923	97.8
Gunma	195	171	7,822	74.9
Saitama	733	694	22,365	64.0
Chiba	626	634	33,009	109.4
Tokyo	1,382	1970	96,514	163.8
Kanagawa	918	327	37,497	88.0
Niigata	225	106	6,823	72.6
Toyama	105	107	3,291	93.3
Ishikawa	114	110	4,509	92.8
Fukui	77	76	3,256	97.3
Yamanashi	82	77	2,504	70.2
Nagano	206	186	6,561	57.6
Gifu	200	279	10,272	104.5
Shizuoka	366	224	15,172	91.8
Aichi	754	689	35,350	119.0
Mie	179	198	5,490	59.5
Shiga	141	150	8,051	100.5
Kyoto	259	136	12,465	96.0
Osaka	881	851	70,134	165.8
Hyogo	548	569	22,062	106.3
Nara	134	156	7,147	102.5
Wakayama	94	132	5,076	92.6
Tottori	56	51	NA	52.9
Shimane	68	78	3,520	90.0
Okayama	190	114	7,285	73.8
Hiroshima	282	182	6,447	83.4
Yamaguchi	137	160	3,854	52.3
Tokushima	74	106	3,337	90.6
Kagawa	96	133	5,673	105.0
Ehime	135	135	6,403	79.9
Kochi	71	74	NA	73.2
Fukuoka	511	293	24,770	99.8
Saga	82	80	NA	49.6
Nagasaki	134	222	6,534	98.7
Kumamoto	176	125	8,725	103.4
Oita	114	169	12,631	187.1
Miyazaki	108	97	3,581	83.3
Kagoshima	161	236	7,198	97.0
Okinawa	145	191	7,902	119.1

TB, tuberculosis; PZA, pyrazinamide; NA, not available.

### 3.2 Regional differences in Pyrazinamide prescriptions

The quantity of PZA prescribed across the prefectures displayed a wide range, from 2,504 to 96,514 g, with a median SCR of 90.0 (range 40.2––187.1, standard deviation (SD) 30.6). The analysis further identified a roughly three-fold difference in the median SCR between the top and bottom prescribing prefectures. Prefectures such as Oita, Osaka, Tokyo, Okinawa, and Aichi were the top prescribers with SCRs of 187.1, 165.8, 163.8, 119.1, and 119.0 respectively, indicative of a higher-than-average prescribing frequency after adjustment for age and gender (Table 1). On the contrary, Akita (SCR 40.2), Yamagata (SCR 44.7), and Saga (SCR 49.6) were among the lowest, suggesting a conservative approach to PZA use, possibly due to previous guidelines or a higher proportion of elderly TB patients. The mean SCR for the five highest prescribing prefectures was 151.0 (SD 30.5), markedly greater than the mean SCR of 47.8 (SD 5.3) for the five lowest, highlighting the need for further investigation into the factors contributing to such regional variations.

### 3.3 Univariate analysis of standardized claim ratio for Pyrazinamide

**Fig. 2:**
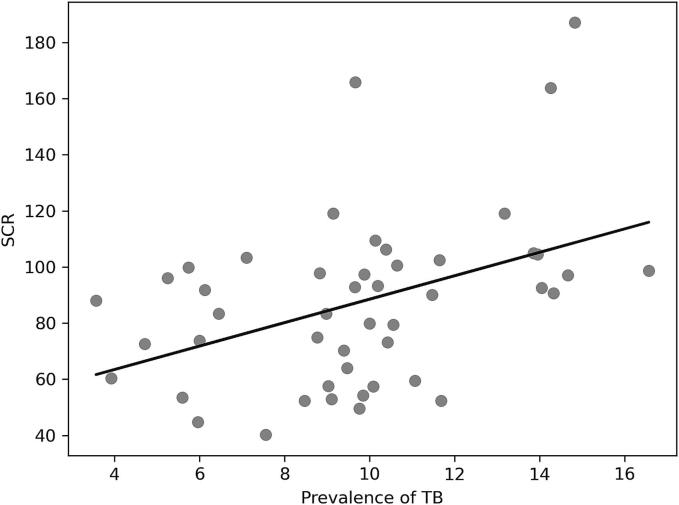

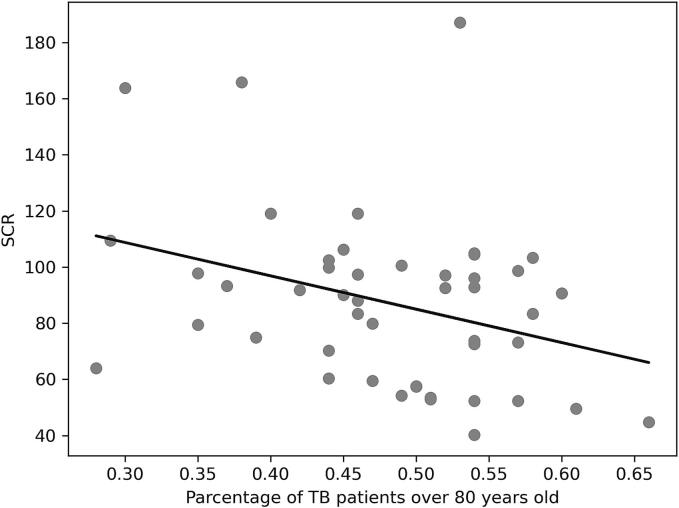

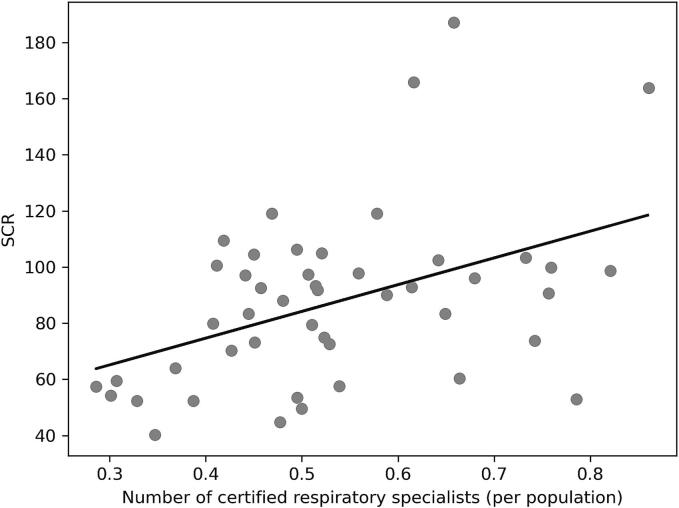

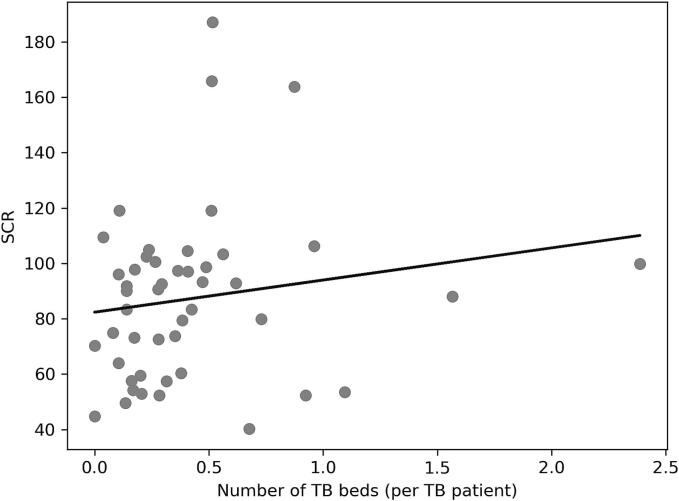

Our univariate analysis, as delineated in Table 2 and depicted graphically in Fig. 2A to 2C, examines the relationship between the SCR for PZA and a series of explanatory variables across Japanese prefectures.

**Table 2:** Correlation between SCR and each factor in 2018.

	*r*	*p*
Prevalence of TB (per 100,000 persons)	0.42	0.0033
Percentage of TB patients over 80 years old	−0.33	0.023
Number of certified respiratory specialists (per population)	0.44	0.0018
Number of certified infectious disease specialists (per population)	0.34	0.021
Number of certified TB specialists (per population)	0.29	0.050
Number of patients with liver disease (per TB patient)	−0.19	0.20
Number of TB beds (per TB patient)	0.16	0.26

TB, tuberculosis; *r*, Pearson's correlation coefficient; *, *p* < 0·05; SCR, standardized claim ratio;

A moderate positive correlation was established between the incidence of TB and the SCR for PZA (r = 0.42, Fig. 2A), indicating that prefectures with higher TB incidence rates tend to prescribe PZA more frequently. This finding aligns with the expected practice pattern where areas with greater TB burdens have a higher utilization of PZA in treatment regimens.

There was a significant inverse relationship between the proportion of elderly TB patients and PZA prescriptions (r = -0.33, Fig. 2B), suggesting that regions with a larger elderly TB patient demographic may prescribe PZA less often, potentially due to heightened vigilance regarding the drug's hepatotoxic risks in older populations.

A robust positive correlation emerged between the number of certified respiratory physicians per population and SCR for PZA (r = 0.44, Fig. 2C). Additionally, the number of certified infectious disease specialists (r = 0.34) and certified TB specialists per population (r = 0.28) both demonstrated positive correlations with PZA SCR, albeit to a slightly lesser degree for TB specialists. This data suggests that the presence of these specialists correlates with a higher propensity to prescribe PZA, possibly reflecting a more guideline-concordant practice pattern. Interestingly, the combination of TB incidence and the number of patients with liver disease per TB patient did not provide a strong correlation (r = -0.69), nor did the combination of infectious disease and TB specialists (r = 0.54).

### 3.4 Multiple regression analysis of standardized claim ratio for Pyrazinamide

Our multiple regression analysis sought to identify the factors that predict the SCR for PZA prescription across Japanese prefectures in 2018, with the findings detailed in Table 3.

**Table 3:** Multiple regression analysis with SCR in 2018 as the objective variable.

Explanatory Variables	B (95 %CI)	*p*	*β*	VIF	*R^2^*
Prevalence of TB (per 100,000 persons)	4.37 (2.10 – 6.65)	0.0004*	0.44	1.06	0.50
Percentage of TB patients over 80 years old	−115.38 (−109.04 – −36.72)	0.0051*	−0.33	1.00	
Number of certified respiratory specialists (per population)	87.61 (38.90 – −136.31)	0·0008*	0.41	1.06	
Number of TB beds (per TB patient)	11.81 (−4.96 – 28.57)	0.16	0.16	1.12	

B, partial regression coefficient; *β*, standardized partial regression coefficient; VIF, variance inflation factor; *R^2^*, coefficient of determination; *, *p* < 0.05; SCR, standardized claim ratio; 95 %CI, 95 % confidence interval; TB, tuberculosis.

The prevalence of TB significantly predicted PZA SCR (B = 4.37, 95 % CI [2.10, 6.65], p < 0.0001), indicating that as the TB prevalence per 100,000 persons increases, so does the ratio of PZA prescriptions. This variable accounted for a substantial 50 % of the variability in SCR (R^2^ = 0.50). The percentage of TB patients over 80 years old was inversely related to the SCR for PZA (B = −115.38, 95 % CI [-109.04, −36.72], p = 0.0051). This indicates a tendency to prescribe PZA less often in prefectures with a higher proportion of elderly TB patients, likely reflecting geriatric considerations and potential drug toxicity concerns.

A positive association was identified between the number of certified respiratory specialists per population and the SCR for PZA (B = 87.61, 95 % CI [38.90, 136.31], p = 0.0008). The presence of these specialists appears to contribute significantly to the likelihood of PZA being prescribed, highlighting the role of specialized care in the management of TB. The number of TB beds per TB patient did not present a significant relationship with PZA SCR (B = 11.81, 95 % CI [-4.96, 28.57], p = 0.16). This finding suggests that the availability of TB-specific hospital beds does not significantly impact PZA prescription rates when other variables are taken into account.

## 4 Discussion

In this study, we analysed regional differences in the SCR of PZA and related factors using data from the NDB database and publicly available SCR data. A roughly threefold difference was observed between the average values of the five prefectures with the highest SCR for PZA and those with the lowest. Multivariable analysis showed that the prevalence of TB, the proportion of elderly patients, and the number of respiratory specialists per population were independently correlated with the SCR of PZA.

The current Japanese tuberculosis treatment guidelines align with WHO and the American Thoracic Society's recommendations, endorsing the HERZ regimen—comprising Isoniazid, Rifampicin, Ethambutol, and Pyrazinamide—as the cornerstone of chemotherapy for TB [6], [7]. Despite the consensus on this regimen, previous iterations of the guidelines in Japan advised a HER regimen, which excludes Pyrazinamide, for specific high-risk patient groups, including those with liver damage, the elderly, and pregnant women [9]. There is a concern that, even following the revision of these guidelines, the persistence of the HER regimen in clinical practice may be erroneously considered as a standard treatment option, potentially due to entrenched clinical habits or misinterpretation of the updated recommendations.

Multivariate analysis identified a significant positive correlation of Pyrazinamide's SCR with the prevalence of TB (*β* = 0.44) and the Number of certified respiratory specialists per capita (*β* = 0.41) in that prefecture (Table 3). This finding suggests that in regions with higher TB prevalence, physicians, including respiratory specialists, are more frequently involved in managing TB patients. This frequent exposure likely enhances their proficiency and confidence in administering treatment and managing side effects, which may result in a higher adherence to guideline-based regimens that include PZA. Some evidence supports the correlation between a physician's expertise and their treatment decisions—Saposnik et al. found that non– multiple sclerosis (MS) specialists were less inclined to intensify treatment in certain MS scenarios [19], and Kingsmore et al. observed that specialists tended to make more appropriate treatment choices in breast cancer care [20]. These observations align with the 'practice-makes-perfect' hypothesis and the concept of 'selective-referral-pattern,' where specialists or reputable institutions provide a higher standard of care. Therefore, in the context of TB treatment, the increased concentration of experienced specialists in high-prevalence areas may explain the higher rates of PZA prescription [21].

The SCR for Pyrazinamide showed a significant negative correlation with the percentage of TB patients over 80 (β = -0.33, Table 3). This finding likely reflects the lasting influence of previous Japanese guidelines that specifically cautioned against PZA use in patients aged ≥ 80 years due to perceived hepatotoxicity risks. [9], [22]. While recent evidence has demonstrated PZA's safety in this age group, and guidelines were updated accordingly in 2018, our findings suggest that this historical age-based recommendation continues to influence prescribing patterns [23], [24]. This persistence of historical practices highlights the challenge of changing established clinical behaviors even after guideline updates. [25]. Recent Japanese studies have provided important evidence regarding PZA safety in elderly populations. Miyazawa et al. demonstrated no significant difference in drug-induced hepatitis between elderly and younger patients receiving PZA-containing regimens [23]. Furthermore, a prospective randomized study by Hagiwara et al. specifically examining patients ≥ 80 years found that PZA-containing regimens did not increase hepatotoxicity compared to non-PZA regimens [25]. More concerning, Hase et al. found that among patients aged ≥ 84 years, those who did not receive PZA showed significantly higher mortality rates compared to those who did (65.8 % vs 36.8 %) [10]. These findings suggest that historical concerns about PZA toxicity in elderly patients may have been overstated, while omitting PZA could potentially compromise treatment outcomes.

No significant correlation was observed between the number of TB isolation beds and PZA-SCR (p = 0.16). This could be attributed to the fact that not all TB patients require isolation beds for treatment, as evidenced by Ministry of Health, Labour and Welfare statistics indicating that only one-third of the new TB cases in 2021 required isolation [26]. Therefore, the impact of patient concentration in hospitals with many TB beds was small overall.

This study has several limitations. First, the weight of the patient, which is necessary to determine the dosage of PZA, could not be obtained. Secondly, the clinical information at the patient level is unclear. Thus, the number of TB patients with liver damage in each prefecture is unknown. Thirdly, the denominator of SCR for sex and age adjustment was based on the population data of that prefecture, and the numerator was based on the number of receipts calculated by medical institutions in the region, which could lead to inaccuracies if there is a large influx or outflow of patients from surrounding areas.

In conclusion, there are regional differences in the prescription of PZA, and the prevalence of TB, the proportion of elderly patients, and the number of respiratory specialists independently influence these regional differences. An increase in respiratory specialists or dissemination of equivalent expertise in TB treatment may contribute to the appropriate prescription of PZA.

## CRediT authorship contribution statement

**Nobuaki Kobayashi:** Writing – review & editing, Writing – original draft, Visualization, Supervision, Project administration, Methodology, Investigation, Formal analysis, Data curation, Conceptualization. **Hiromi Matsumoto:** Writing – original draft, Methodology, Investigation, Formal analysis, Data curation. **Takeshi Kaneko:** Writing – review & editing, Supervision.

## Declaration of competing interest

The authors declare that they have no known competing financial interests or personal relationships that could have appeared to influence the work reported in this paper.
